# Antivirulence Bispecific Monoclonal Antibody-Mediated Protection against Pseudomonas aeruginosa Ventilator-Associated Pneumonia in a Rabbit Model

**DOI:** 10.1128/aac.02022-21

**Published:** 2022-02-15

**Authors:** Fábio Aguiar-Alves, Hoan N. Le, Vuvi G. Tran, Emmanuelle Gras, Trang T. T. Vu, Oliver X. Dong, Josiane Silva Quetz, Lily I. Cheng, Li Yu, Bret R. Sellman, Charles K. Stover, Antonio DiGiandomenico, Binh An Diep

**Affiliations:** a Division of HIV, Infectious Diseases, and Global Medicine, Department of Medicine, University of California, San Francisco, San Francisco, California, USA; b Pathology Program, Fluminense Federal University, Niterói, Rio de Janeiro, Brazil; c François Rabelais University, Tours, France; d Discovery Microbiome, Microbial Sciences, Biopharmaceuticals R&D, AstraZeneca, Gaithersburg, Maryland, USA; e Research and Early Development, Oncology R&D, AstraZeneca, Gaithersburg, Maryland, USA

**Keywords:** *Pseudomonas aeruginosa*, ventilator-associated pneumonia, immunotherapy, pneumonia

## Abstract

Ventilator-associated pneumonia is an important clinical manifestation of the nosocomial pathogen Pseudomonas aeruginosa. We characterized the correlates of protection with MEDI3902, a bispecific human IgG1 monoclonal antibody that targets the P. aeruginosa type 3 secretion system PcrV protein and the Psl exopolysaccharide, in a rabbit model of ventilator-associated pneumonia using lung-protective, low-tidal-volume mechanical ventilation. Rabbits infused with MEDI3902 prophylactically were protected, whereas those pretreated with irrelevant isotype-matched control IgG (c-IgG) succumbed between 12 and 44 h postinfection (100% survival [8/8 rabbits] versus 0% survival [8/8 rabbits]; *P* < 0.01 by log rank test). Lungs from rabbits pretreated with c-IgG, but not those pretreated with MEDI3902, had bilateral, multifocal areas of marked necrosis, hemorrhage, neutrophilic inflammatory infiltrate, and diffuse fibrinous edema in alveolar spaces. All rabbits pretreated with c-IgG developed worsening bacteremia that peaked at the time of death, whereas only 38% of rabbits pretreated with MEDI3902 (3/8 rabbits) developed such high-grade bacteremia (two-sided Fisher’s exact test, *P *= 0.026). Biomarkers associated with acute respiratory distress syndrome were evaluated longitudinally in blood samples collected every 2 to 4 h to assess systemic pathophysiological changes in rabbits pretreated with MEDI3902 or c-IgG. Biomarkers were sharply increased or decreased in rabbits pretreated with c-IgG but not those pretreated with MEDI3902, including the ratio of arterial oxygen partial pressure to the fraction of inspired oxygen of <300, hypercapnia or hypocapnia, severe lactic acidosis, leukopenia, and neutropenia. Cytokines and chemokines associated with acute respiratory distress syndrome were significantly downregulated in lungs from rabbits pretreated with MEDI3902, compared with c-IgG. These results suggest that MEDI3902 prophylaxis could have potential clinical utility for decreasing the severity of P. aeruginosa ventilator-associated pneumonia.

## TEXT

Ventilator-associated pneumonia is one of the most frequent nosocomial infections, affecting 1 in 3 patients on mechanical ventilation (1). One of the pathogens, Pseudomonas aeruginosa, is recognized as the most common agent in nosocomial infections (2), in addition to causing approximately 21% to 24% of all ventilator-associated pneumonia cases in the United States (3). Antibiotics have always been the main treatment for bacterial infections, but the current alarming rate of antibiotic resistance, particularly among P. aeruginosa and the rest of the so-called ESKAPE (Enterococcus faecium, Staphylococcus aureus, Klebsiella pneumoniae, Acinetobacter baumannii, Pseudomonas aeruginosa, and Enterobacter species) pathogens, poses a significant challenge to modern medicine (4). Although general infection control strategies exist to decrease the incidence of ventilator-associated pneumonia among hospitalized patients, these infections are associated with significant mortality and morbidity rates, increased intensive care unit (ICU) utilization, and increased hospital length of stay, resulting in a substantial economic burden (5, 6).

MEDI3902 is a multimechanistic bivalent and bispecific monoclonal antibody (MAb) targeting Psl exopolysaccharide and PcrV (7), and it recently completed evaluation for the prevention of pneumonia in ventilated patients colonized with P. aeruginosa (Effort to Prevent Nosocomial Pneumonia Caused by P. aeruginosa in Mechanically Ventilated Subjects [EVADE] study [ClinicalTrials registration number NCT02696902]). While that proof-of-concept study did not achieve its primary endpoint, evidence of a responder population was identified, suggesting that this molecule has a path forward for continued clinical development (8). Both Psl and PcrV virulence determinants are highly conserved among P. aeruginosa clinical isolates (9). MEDI3902 mediates at least three mechanisms of action, namely, anti-Psl activity promotes complement-dependent opsonophagocytic killing and prevents P. aeruginosa attachment to host epithelial cells, and anti-PcrV prevents killing of host cells via the type 3 secretion system (10, 11). Combining each MAb specificity into MEDI3902 was shown to mediate synergistic protective activity in mice, compared to individual parental MAbs (7, 12).

The objective of this study is to compare the protective efficacy of MEDI3902 and an irrelevant isotype-matched control IgG (c-IgG) in a recently described rabbit model of ventilator-associated pneumonia (13). Although nonventilated mouse and rabbit pneumonia models were used previously to demonstrate the efficacy of MEDI3902 (7, 14–16), a limitation of those studies is the lack of mechanical ventilation, which is a major predisposing risk factor for P. aeruginosa ventilator-associated pneumonia (17–19). To better mimic lung-protective mechanical ventilation strategies used clinically (20), our rabbits were ventilated with a low tidal volume of 6 to 7 mL/kg and positive end-expiratory pressure (PEEP) of 6 cm H_2_O (13), which were not employed in previous rabbit models of ventilator-associated pneumonia (21, 22). In our rabbit model of ventilator-associated pneumonia, MEDI3902 prophylaxis protected against lethal acute lung injury and inflammation.

## RESULTS

### MEDI3902 protected against P. aeruginosa ventilator-associated pneumonia.

**FIG 1:**
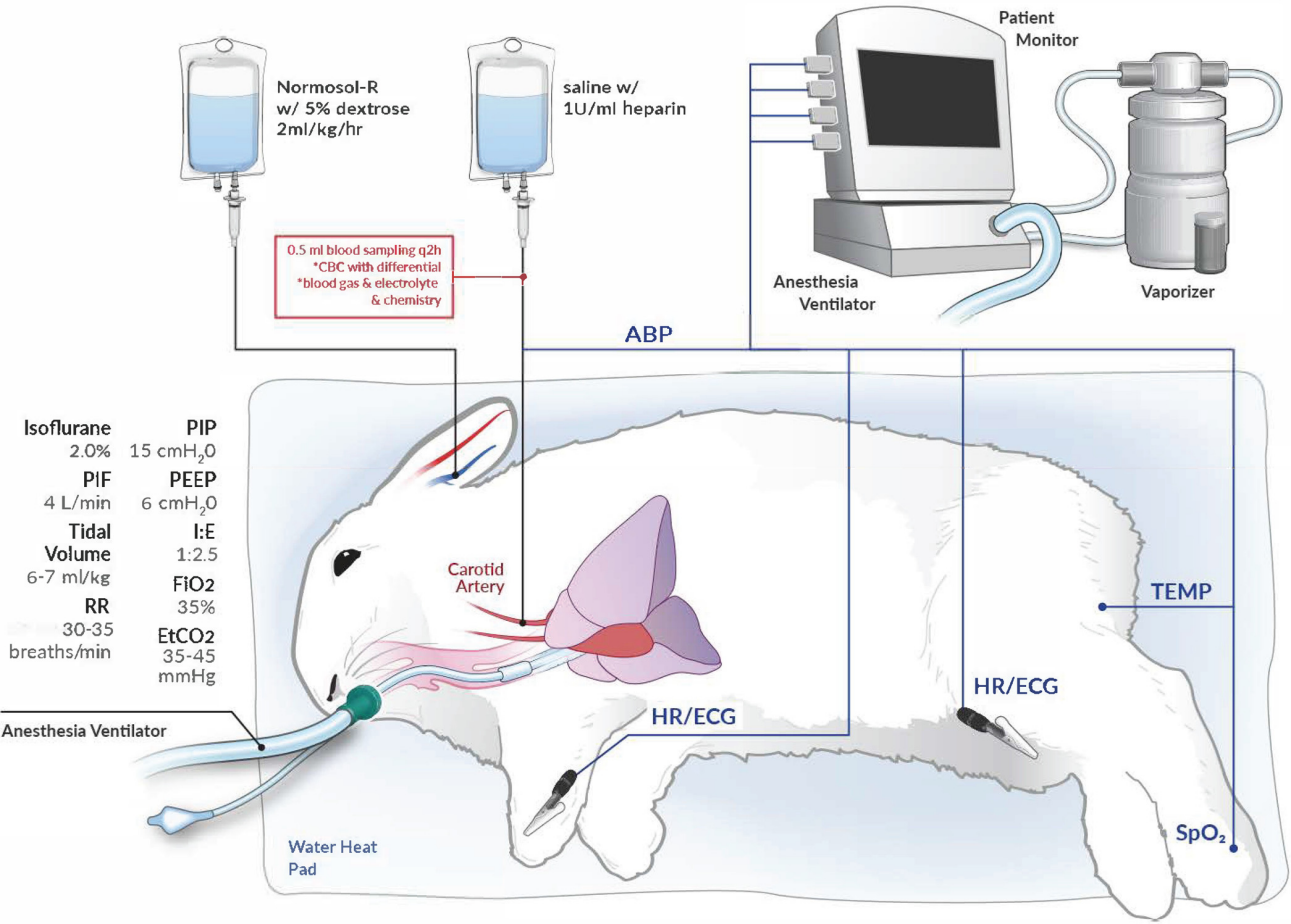
Schematic of the experimental setup for a rabbit model of ventilator-associated pneumonia. Rabbits were intubated and mechanically ventilated with a PIP of 15 cm H_2_O, PEEP of 6 cm H_2_O, peak inspiratory flow (PIF) of 4 L/min, and FiO_2_ of 0.35, with 2.0% isoflurane to maintain general anesthesia. The respiratory rate (RR) was adjusted to 30 to 35 breaths/min to achieve end-tidal CO_2_ (EtCO_2_) of 35 to 45 mm Hg. The mechanical ventilation parameters resulted in a low tidal volume of 6 to 7 mL/kg and an inspiratory/expiratory ratio (I:E) of 1:2.5. The carotid artery was cannulated for serial blood sampling and arterial blood pressure (ABP) monitoring. The marginal ear vein was cannulated for infusion of Normosol-R with 5% dextrose for fluid maintenance. The patient monitor was used for continuous monitoring of heart rate (HR), electrocardiogram (ECG), rectal temperature, and peripheral capillary oxygen saturation (SpO_2_).

**FIG 2:**
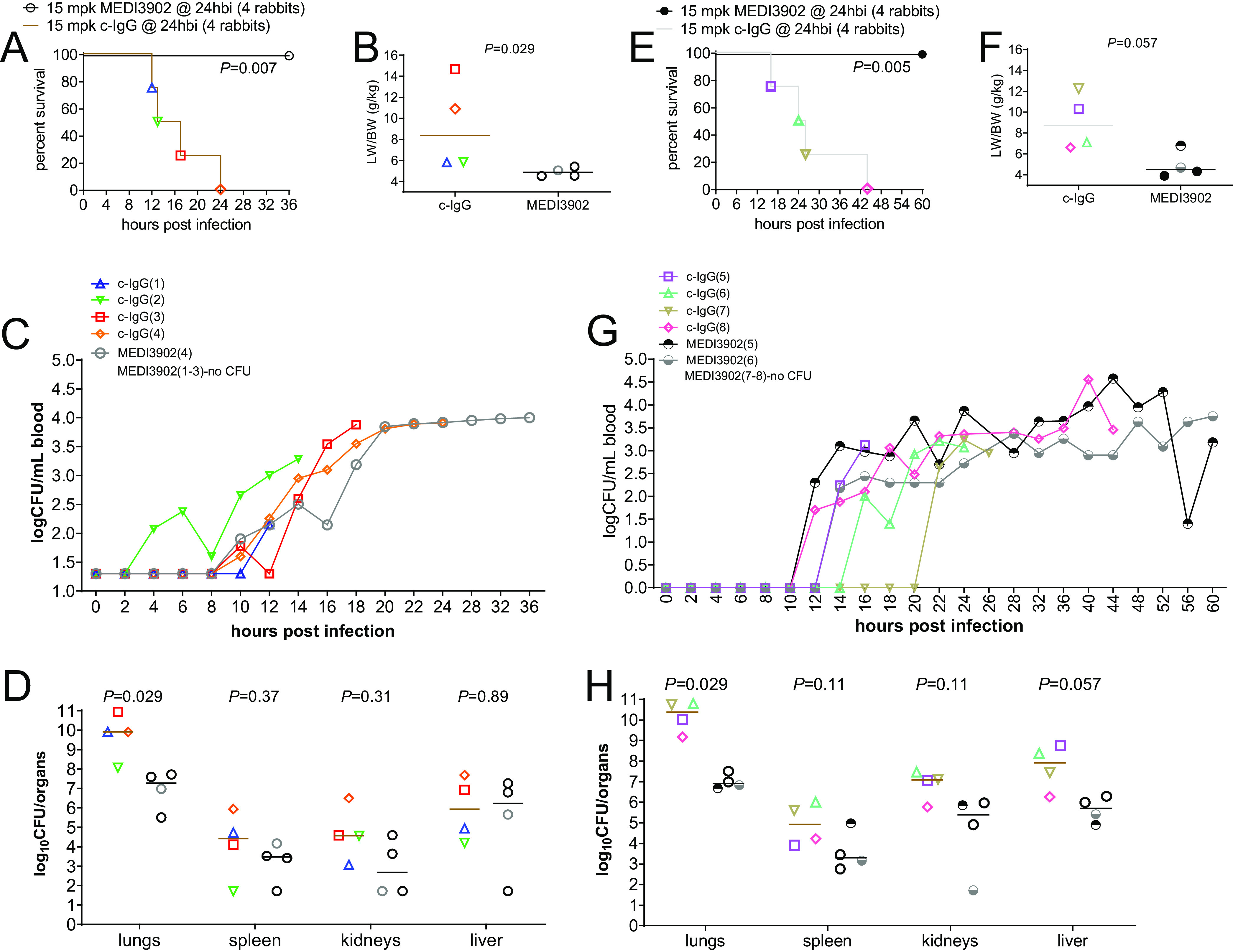
MEDI3902 prophylaxis improved survival and reduced bacterial burden in a rabbit model of ventilator-associated pneumonia. Kaplan-Meier survival curves (A and E), LW/BW ratios (B and F), log_10_CFU per milliliter of blood (C and G), and log_10_CFU per organ (D and H) for rabbits treated intravenously with 15 mg/kg MEDI3902 or 15 mg/kg c-IgG 24 h before infection with P. aeruginosa strain 6077 and monitored for 36 h (study 1) (A to D) or 60 h (study 2) (E to H) were compared. Bars indicate the medians for all treatment groups. The LW/BW ratio and log_10_CFU per organ for rabbits pretreated with MEDI3902 were compared to values for rabbits pretreated with c-IgG by nonparametric Mann-Whitney *U* test.

Rabbits were intubated and mechanically ventilated with lung-protective strategies (20), namely, low tidal volume of 6 to 7 mL/kg, PEEP of 6 cm H_2_O, and fraction of inspired oxygen (FiO_2_) of 0.35, which achieved an end-tidal CO_2_ goal of 35 to 45 mm Hg at baseline before infection (Fig. 1). To establish ventilator-associated pneumonia, cytotoxic P. aeruginosa strain 6077 was instilled directly into the lungs 2 to 3 h after the start of ventilation, and then the rabbits were monitored for 36 h postinfection (hpi) (study 1) or 60 hpi (study 2). All 8 rabbits that were pretreated with c-IgG died between 12 and 44 hpi, whereas all 8 of those pretreated with MEDI3902 survived to 36 h or 60 hpi, when they were euthanized (log rank test, *P *< 0.01) (Fig. 2A and E).

Of the rabbits that were pretreated with c-IgG, 50% (4/8 rabbits) had lung weight (LW) to body weight (BW) ratios exceeding 10 g/kg (Fig. 2B and F), a pulmonary edema severity threshold value that we have found to be associated with profound respiratory failure and death in rabbits (16). The other rabbits that were pretreated with c-IgG (4/8 rabbits) or MEDI3902 (8/8 rabbits) had LW/BW ratios ranging from 4 to 7 g/kg, indicating minimal to moderate pulmonary edema severity (Fig. 2B and F).

**FIG 3:**
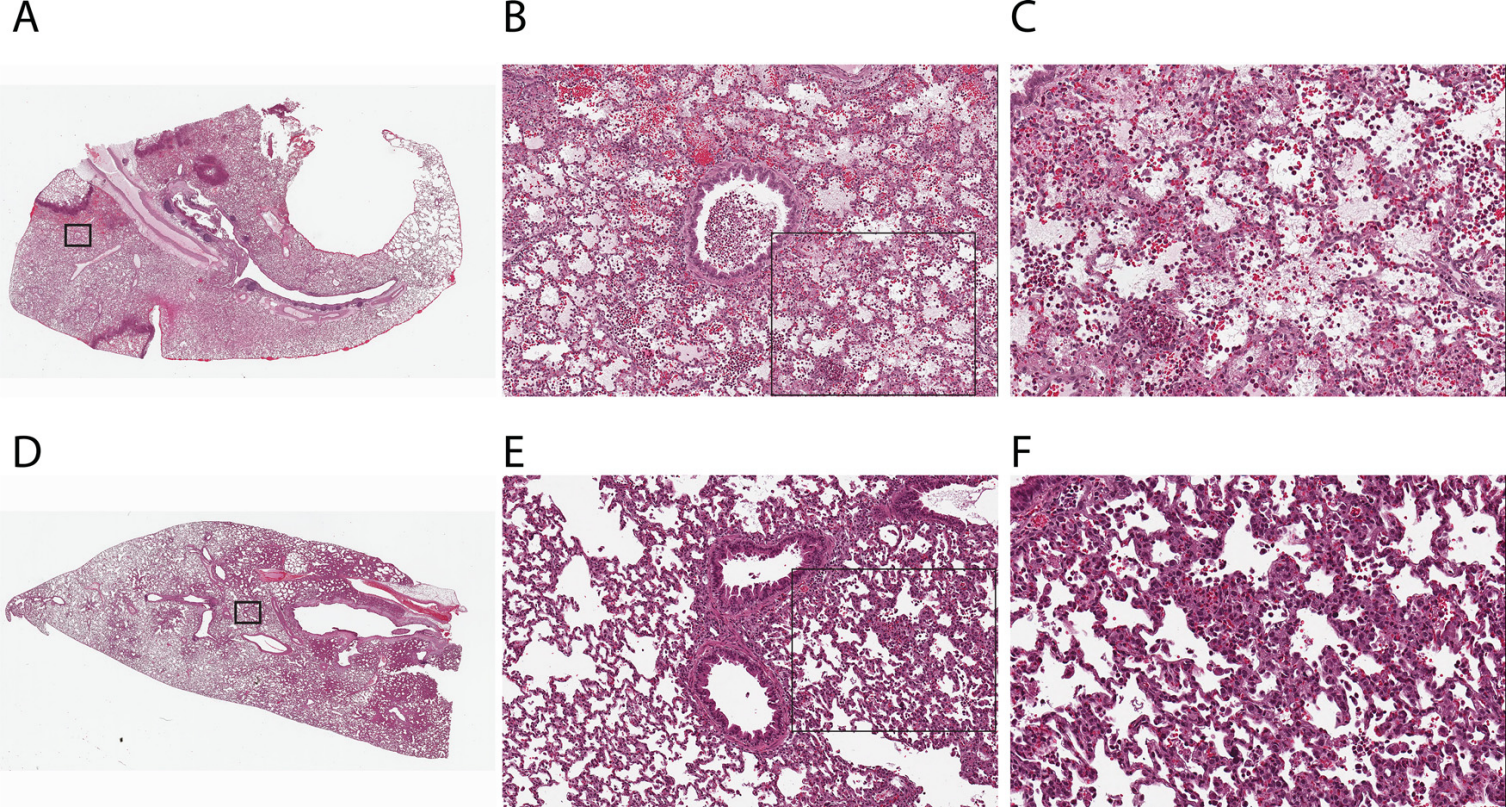
Histology showed reduced acute lung injury and inflammation for lungs harvested from rabbits pretreated with MEDI3902, compared to those pretreated with c-IgG. Photomicrographs of hematoxylin- and eosin-stained representative lung sections from the rabbit model of ventilator-associated pneumonia are shown for animals pretreated intravenously with 15 mg/kg of c-IgG (A to C) or MEDI3902 (D to F). (A) Prophylaxis with c-IgG results in multifocal areas of pulmonary necrosis and hemorrhage (×2 magnification). (B) Inset, bronchioles contain inflammatory cellular debris (×10 magnification). (C) Inset, alveolar septal necrosis and alveolar spaces are filled with fibrinous edema, hemorrhage, and abundant inflammatory cells (×20 magnification). (D) Prophylaxis with MEDI3902 demonstrated mild to patchy moderate infiltrate of viable inflammatory cells (×2 magnification). (E) Inset, multifocal areas of mild alveolar septal thickening and small aggregates of inflammatory cells within alveolar spaces are evident. Bronchioles are within normal limits (×10 magnification). (F) Inset, alveolar spaces contain primarily heterophils admixed with macrophages (×20 magnification).

Macroscopically, gross images of lungs from rabbits pretreated with c-IgG, but not those pretreated with MEDI3902, showed extensive areas of lung hemorrhage and necrosis (see Fig. S1 in the supplemental material). Microscopically, lungs from rabbits pretreated with c-IgG demonstrated multifocal areas of marked necrosis, hemorrhage, neutrophilic inflammatory infiltrate, and diffuse fibrinous edema in alveolar spaces (Fig. 3A and C); bronchioles often contained accumulations of degenerate inflammatory cells within the lumen (Fig. 3B). In contrast, lungs from rabbits pretreated with MEDI3902 showed multifocal mild thickening of alveolar walls and minimal to mild infiltrates of inflammatory cells within alveoli (Fig. 3D to F).

### MEDI3902 protected against P. aeruginosa dissemination from the lungs.

All rabbits that were pretreated with c-IgG (8/8 rabbits) developed progressively worsening bacteremia, which peaked at the time of death, whereas only 38% of rabbits pretreated with MEDI3902 (3/8 rabbits), denoted MEDI3902(4), MEDI3902(5), and MEDI3902(6), developed high-grade bacteremia (two-sided Fisher’s exact test, *P *= 0.026) (Fig. 2C and G). Bacterial counts in lungs were significantly reduced in rabbits pretreated with MEDI3902, compared to those pretreated with c-IgG, although between-group differences were lesser in spleen, kidney, and liver samples (Fig. 2D and H).

### ARDS biomarkers correlated with disease outcomes.

**FIG 4:**
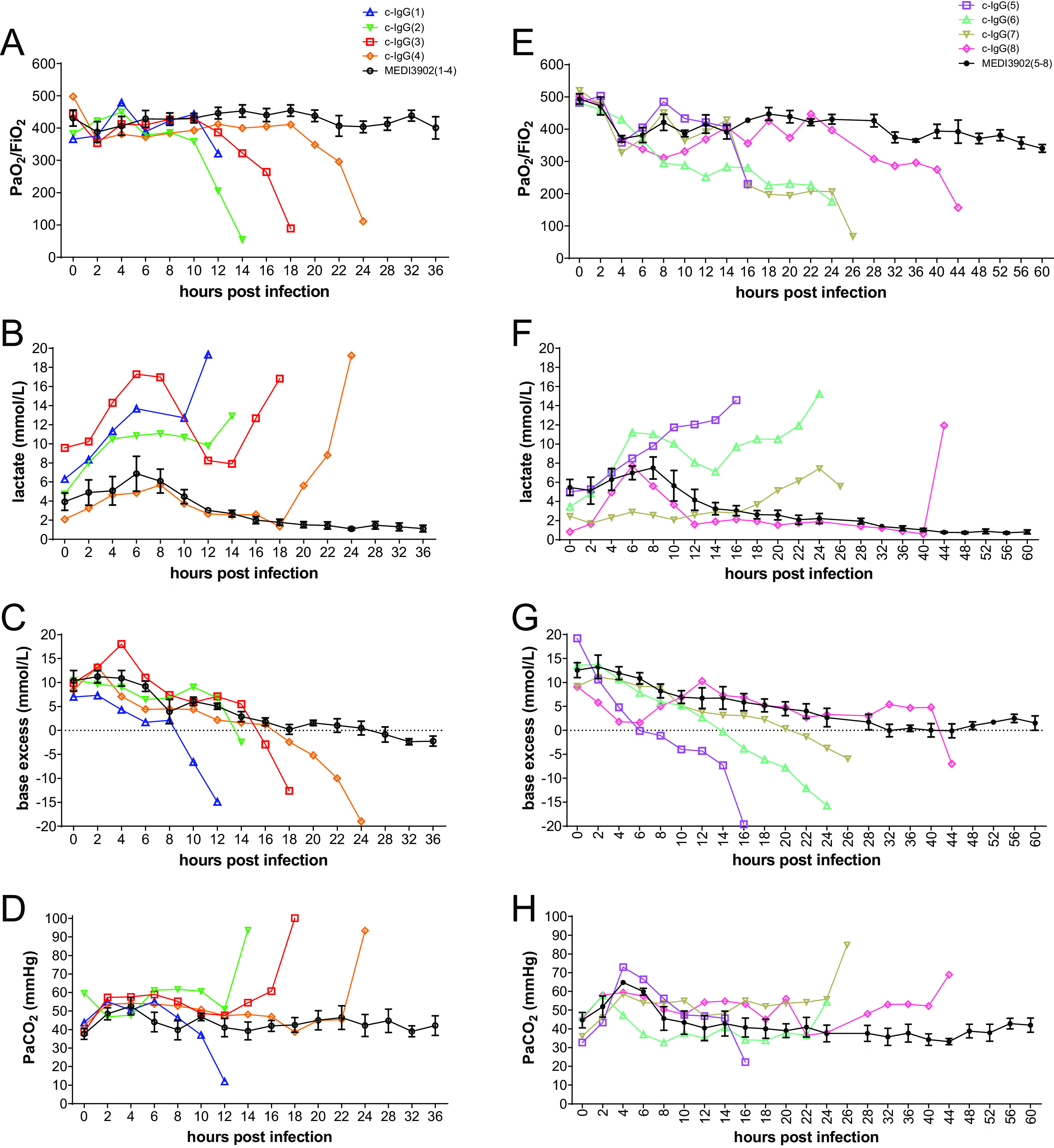
Longitudinal analysis of biomarkers associated with ARDS. PaO_2_/FiO_2_ (A and E), lactate levels (B and F), base excess (C and G), and PaCO_2_ (D and H) were determined using arterial blood samples taken every 2 h for the first 24 h and every 4 h thereafter until survivors were euthanized at 36 hpi (study 1) (A to D) or 60 h (study 2) (E to H). Additional parameters, including electrolyte and glucose levels, are shown in Fig. S3 in the supplemental material.

To systematically assess biomarker changes associated with survival outcomes for rabbits pretreated with c-IgG or MEDI3902, we collected arterial blood samples from each rabbit every 2 h for the first 24 h and every 4 h thereafter, for detailed longitudinal analyses. All rabbits pretreated with MEDI3902 (8/8 rabbits) showed little change in the arterial oxygen partial pressure (PaO_2_)/FiO_2_ ratio from preinfection baseline throughout the 36-h or 60-h monitoring period, whereas 75% of those pretreated with c-IgG (6/8 rabbits) developed moderate to severe acute respiratory distress syndrome (ARDS), with PaO_2_/FiO_2_ ratios of <200 at the time preceding death (Fig. 4A and E). Death in rabbits pretreated with c-IgG was associated with marked increase in lactate levels (Fig. 4B and F) and decline in base excess (Fig. 4C and G).

Although arterial carbon dioxide partial pressure (PaCO_2_) showed little change from preinfection baseline for all rabbits pretreated with MEDI3902 (8/8 rabbits), 63% of rabbits pretreated with c-IgG (5/8 rabbits) developed hypercapnia and 25% (2/8 rabbits) developed hypocapnia (Fig. 4D and H). Because hypocapnia is a feature of severe Gram-negative septic shock (23), the two rabbits, denoted c-IgG(1) and c-IgG(5), with this preterminal condition also developed multiple organ dysfunction, as evidenced by >2-fold increases from preinfection baseline in levels of creatinine (a marker of acute kidney injury), amylase (a marker of acute pancreatic injury), and/or alanine aminotransferase (a marker of acute liver injury) (see Fig. S2).

Electrolyte and glucose abnormalities, including hyperkalemia, hyponatremia, and hyperglycemia/hypoglycemia, were noted in a subset of rabbits pretreated with c-IgG but not those pretreated with MEDI3902 (see Fig. S3).

**FIG 5:**
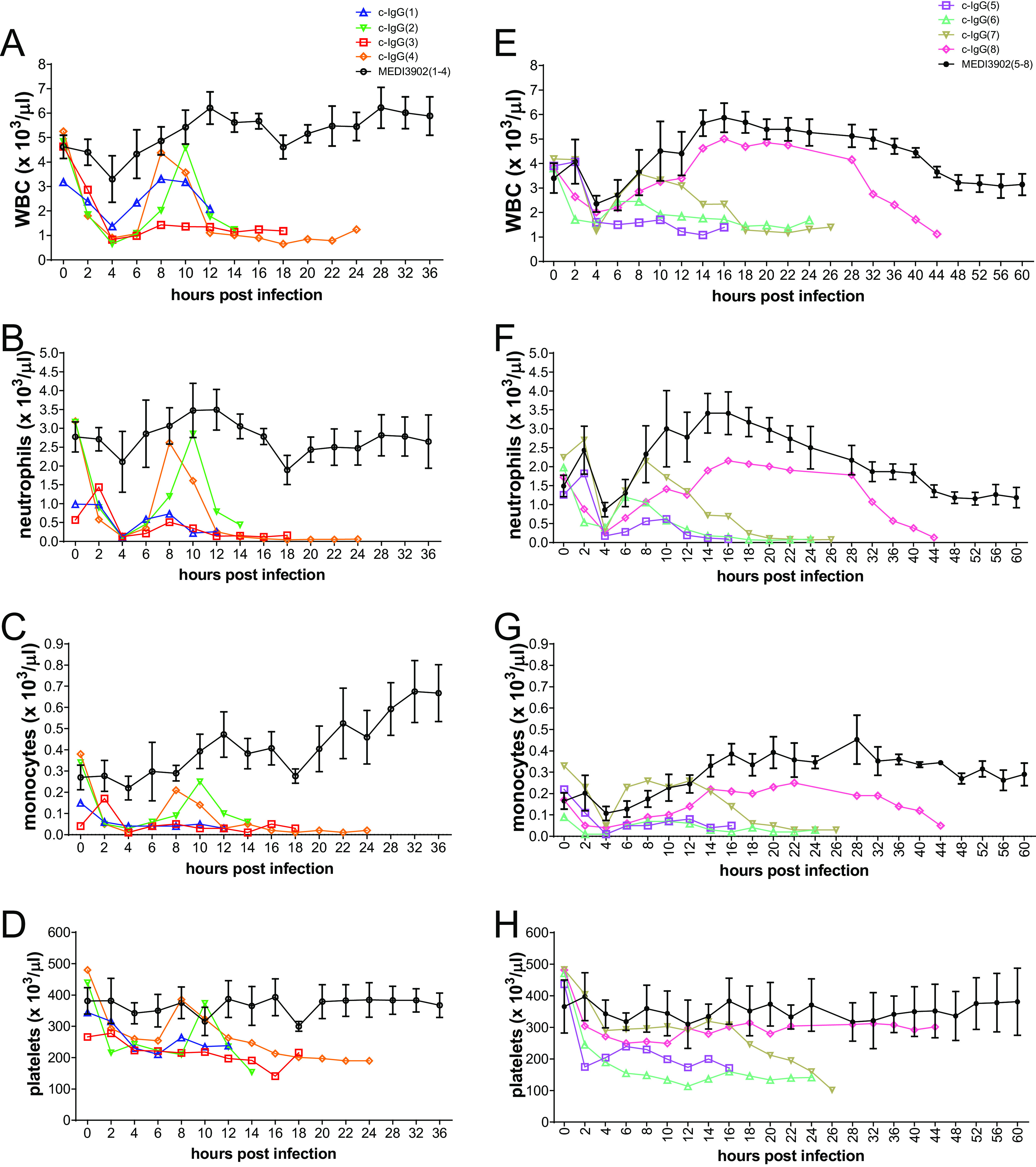
Longitudinal analysis of complete blood counts in rabbits pretreated with MEDI3902 versus c-IgG. Counts of WBCs (A and E), neutrophils (B and F), monocytes (C and G), and platelets (D and H) were determined using arterial blood samples taken every 2 h for the first 24 h and every 4 h thereafter until survivors were euthanized at 36 hpi (study 1) (A to D) or 60 h (study 2) (E to H). Additional hematological parameters are shown in Fig. S4 in the supplemental material.

Rabbits pretreated with c-IgG developed severe leukopenia, neutropenia, and monocytopenia, as well as modest decreases in platelet counts, whereas white blood cell (WBC) counts remained little changed from preinfection baseline for those pretreated with MEDI3902 (Fig. 5; also see Fig. S4).

### MEDI3902 prophylaxis protected against acute lung inflammation.

Comparative analysis of the acute lung inflammatory response was performed with RNAlater-preserved lung samples from rabbits pretreated with MEDI3902, compared to those pretreated with c-IgG, using quantitative reverse transcriptase-PCR (qRT-PCR) for analysis of differential expression of 84 inflammatory cytokines and receptors (16). Notably, interleukin-6 (IL-6), IL-23α, IL-1α, IL-1β, IL-8, IL-10, IL-20, monocyte chemoattractant protein 1 (MCP-1) (C-C motif chemokine ligand 2 [CCL2]), leukemia inhibitory factor (LIF), granulocyte-macrophage colony-stimulating factor (CSF2), macrophage inflammatory protein 1β (CCL4), tumor necrosis factor alpha (TNF-α), and oncostatin M (OSM), were downregulated, while the CXCR3 chemokine receptor was upregulated, in rabbits pretreated with MEDI3902, compared to those pretreated with c-IgG (Table 1). The overall reduction in expression of these inflammatory cytokines and chemokines, which are important in host immune activation from pathogen recognition, endothelial activation, and neutrophil recruitment to the lung (24–31), is consistent with MEDI3902 prophylaxis limiting P. aeruginosa-induced acute lung inflammation, whereas c-IgG prophylaxis had no protective effect, resulting in massive inflammatory infiltrate, acute lung injury, and death.

**TABLE 1:** MEDI3902 prophylaxis reduced acute lung inflammation

Gene^*a*^	36-h study (MEDI3902 vs c-IgG)		60-h study (MEDI3902 vs c-IgG)	
	Fold change (95% CI)	*P*	Fold change (95% CI)	*P*
*IL6*	−957 (−15,881 to −58)	0.006	−1,312 (−12,676 to −136)	0.001
*CCL2*	−92 (−356 to −24)	0.003	−52 (−233 to −11)	0.002
*LIF*	−87 (−254 to −30)	0.001	−147 (−495 to −44)	0.001
*IL23A*	−78 (−278 to −22)	0.001	−40 (−141 to −11)	0.001
*IL1A*	−60 (−414 to −9)	0.009	−54 (−156 to −19)	0.001
*IL8*	−36 (−185 to −7)	0.007	−31 (−113 to −9)	0.001
*CCL4*	−25.2 (−292.7 to −2.2)	0.027	−15.7 (−54. 9 to −4.5)	0.002
*TNF*	−23.8 (−68.5 to −8.3)	0.002	−11.7 (−33.5 to −4.1)	0.001
*IL10*	−17.5 (−64.6 to −4.7)	0.009	−16.2 (−103.3 to −2.5)	0.015
*CSF2*	−16.5 (−43.5 to −6.3)	0.002	−7.1 (−21.2 to −2.4)	0.004
*IL1B*	−13.5 (−72.5 to −2.5)	0.017	−16.8 (−44.1 to −6.4)	0.002
*IL20*	−12.6 (−38.0 to −4.1)	0.005	−28.4 (−177. 9 to −4.5)	0.004
*OSM*	−6.2 (−16.8 to −2.3)	0.009	−10.4 (−23.7 to −4.6)	0.001
*CXCR3*	5.3 (2.7 to 10.3)	0.002	6.8 (2.9 to 15.9)	0.002

a Inflammatory genes or their receptors that were downregulated or upregulated at least 2-fold when survivor rabbits pretreated with MEDI3902 and euthanized at 36 hpi or 60 hpi were compared to nonsurvivors pretreated with c-IgG.

## DISCUSSION

Here, we showed that MEDI3902 prophylaxis protected against acute lung injury and inflammation in a clinically relevant rabbit model of ventilator-associated pneumonia with the multidrug-resistant, ExoU-producing P. aeruginosa strain 6077. Inasmuch as ExoU is critical for P. aeruginosa to cause lethal pneumonia in a nonventilated mouse model and a ventilated rabbit model (11, 22), our efficacy results with MEDI3902 are consistent with its specific activity in blocking the type III needle-tip protein PcrV (11) from secretion of ExoU and thus inhibiting the toxin from causing pulmonary epithelial and endothelial cellular damage (32, 33). Despite the use of lung-protective low-tidal-volume mechanical ventilation (Fig. 1), rabbits pretreated with c-IgG, but not those pretreated with MEDI3902, developed PaO_2_/FiO_2_ ratios of <300 mm Hg (Fig. 2), a hallmark clinical feature of ARDS (34). Our longitudinal analysis of biomarkers in serial blood samples in the rabbit model of ventilator-associated pneumonia illustrated the remarkable capacity of this model to recapitulate the chronology of clinical ARDS, i.e., increasing severity of neutropenia in peripheral blood that was accompanied by widespread neutrophil influx into the alveolar space, deteriorating hypoxemia, and hypercapnia with lactic acidosis in most rabbits pretreated with c-IgG. Because MEDI3902 protected against P. aeruginosa*-*induced damage to the alveolar-endothelial barrier, it also reduced dissemination of bacteria into the blood (Fig. 2C and G).

Two of eight rabbits (25%) pretreated with c-IgG [i.e., c-IgG(1) and c-IgG(5)], but none of those pretreated with MEDI3902, developed septic shock-like syndrome, including severe hypotension, hypocapnia, and multiple organ dysfunction (Fig. 4D and H; also see Fig. S2 in the supplemental material). The presence of septic shock in a subset of our rabbits with ventilator-associated pneumonia reproduced a hallmark clinical feature in which ∼25% of patients with ventilator-associated pneumonia developed septic shock (35). Septic shock could be reproduced in 100% of animals used in a previously published rabbit model of ventilator-associated pneumonia that employed a lung-damaging tidal volume of 20 mL/kg (21, 22), although that experimental strategy does not reproduce the lung-protective low tidal volume used clinically (20) and in our rabbit model described here (Fig. 1).

MEDI3902 also protected against acute lung inflammation (Table 1), thereby reducing the massive neutrophilic inflammatory infiltrate that would otherwise contribute to the ARDS pathogenesis (Fig. 3 and 4). These MEDI3902-mediated effects enhanced survival outcomes in the rabbit model of ventilator-associated pneumonia (Fig. 2A and C).

The protective efficacy of MEDI3902 prophylaxis in the rabbit model of ventilator-associated pneumonia is consistent with our recent findings that MEDI3902 prophylaxis also conferred protection in a rabbit model of nonventilated acute pneumonia (16). Despite the use of the same P. aeruginosa strain 6077 in both rabbit models, MEDI3902 prophylaxis resulted in greater protection against acute lung injury in the rabbit model of ventilator-associated pneumonia, as evidenced by a LW/BW ratio of 4.91 g/kg for ventilated lungs versus 8.88 g/kg for nonventilated lungs from rabbits pretreated with MEDI3902 (Fig. 2B and F here versus Fig. 1B in reference 16). This could be due to the additional benefits of lung-protective mechanical ventilation involving a low tidal volume of 6 to 7 mL/kg and a PEEP of 6 cm H_2_O (Fig. 1), which were proven to decrease mortality rates and to improve clinical outcomes in the landmark ARDS Network study (20).

Expression of cytokines and chemokines in the rabbit lungs was markedly and significantly attenuated by MEDI3902 prophylaxis, compared to c-IgG prophylaxis (Table 1). IL-6 expression was the most prominently decreased (>900-fold) in rabbits pretreated with MEDI3902, compared to those pretreated with c-IgG. CCL2, LIF, IL-23A, IL-1β, and CXCL8 were also highly decreased >30-fold (Table 1). All of these cytokines have pivotal roles in inducing massive inflammatory cell infiltration, resulting in an uncontrolled inflammatory response (24–31). Interestingly, when inflammatory gene expression in the rabbit model of ventilator-associated pneumonia in the present work was compared to recently published findings for a rabbit model of nonventilated acute pneumonia, MEDI3902 prophylaxis showed greater reductions in inflammatory gene expression in the ventilated lungs than in the nonventilated lungs (Table 1 here versus Table 1 in reference 16), which could be due to the additional benefits of lung-protective low-tidal-volume mechanical ventilation. Alternatively, differences in gene expression between the two rabbit models could be due to the fact that ventilated lungs were harvested at later stages of infection than nonventilated lungs (i.e., 23 hpi for ventilated lungs versus 10 hpi for nonventilated lungs from rabbits pretreated with c-IgG [16]). Despite greater reduction in gene expression with MEDI3902 in the rabbit model of ventilator-associated pneumonia, the reduced inflammatory markers were predominantly the same for the two rabbit models, with only a few exceptions, i.e., CSF2, TNF, and IL-20 were downregulated in ventilated lungs but unchanged in nonventilated lungs after MEDI3902 prophylaxis, and CXCR3 was upregulated only in ventilated lungs and not in nonventilated lungs after MEDI3902 prophylaxis (Table 1 here versus Table 1 in reference 16). The smaller effect of CXCR3 upregulation may be due to a possible increase in CXCR3-bearing cells such as natural killer T cells (NKT cells), which have an established role in clearing microorganisms from the airways (36, 37). Further studies will be needed to clarify the relevance of this specific upregulated gene expression to the overall benefits of MEDI3902 prophylaxis in the ventilator-associated pneumonia context.

Our study has limitations. First, the efficacy of MEDI3902 was evaluated in the present study only as preexposure prophylaxis. However, this rabbit model, which was shown recently to be useful for postexposure treatment studies with ICU supportive care (e.g., fluid challenge and vasopressor) and a humanized dosing regimen of standard-of-care antibiotic, will allow preclinical testing of MEDI3902 in a manner that mimics how this novel molecule could be used clinically to treat ventilator-associated pneumonia (13). Second, although we used low-tidal-volume mechanical ventilation in the present study, the rabbits were ventilated with nonheated, nonhumidified air, which may contribute to lung injury (data not shown) (38, 39). Because humidification of inhaled gas is a standard of care, we recently introduced a refinement for this rabbit model to include active heater humidifiers to compensate for the lack of natural humidification mechanisms in the intubated rabbits (13). Notwithstanding the lack of humidification, MEDI3902 conferred significant protection against P. aeruginosa in our rabbit model of ventilator-associated pneumonia. Third, although arterial blood pressure was continuously monitored, it was not recorded due to lack of equipment at that time, resulting in a missed opportunity to better characterize MEDI3902-mediated protection in the rabbit model. Consistent with our recent natural history studies to validate the rabbit model of ventilator-associated pneumonia (13), we did notice that rabbits pretreated with c-IgG here, but not those pretreated with MEDI3902, all developed severe hypotension in the hours preceding death. Severe hypotension in this rabbit model is nonresponsive to fluid challenge alone, requiring vasopressor to ensure adequate blood pressure and oxygen delivery to prevent multiple organ dysfunction (13), which was also observed in a subset of rabbits used here (see Fig. S2).

Taken together, our results showed here that MEDI3902 prophylaxis conferred significant protection in a clinically relevant rabbit model of ventilator-associated pneumonia, supporting the continued development of this molecule for reducing the severity of P. aeruginosa-induced ventilator-associated pneumonia.

## MATERIALS AND METHODS

### Bacterial strain and growth conditions.

P. aeruginosa strain 6077, a cytotoxic strain that expresses the type III secreted toxins ExoU, ExoT, and ExoY, was used in the rabbit model of ventilator-associated pneumonia. Strain 6077 was a gift from Joanna Goldberg (Emory University). Strain 6077 was prepared as described previously for infection of rabbits (15, 16). In brief, an overnight culture of strain 6077 was grown in 12 mL of tryptic soy broth (TSB) in a 50-mL vented-cap tube at 37°C for 16 h, with shaking at 150 rpm. The overnight culture (60 μl) was then transferred to 12 mL of fresh TSB and incubated at 37°C for 14 h, with shaking at 150 rpm, to an optical density at 600 nm (OD_600_) of approximately 1.7. Bacteria were collected by centrifugation at 16°C, washed once, and then resuspended in lactated Ringer’s solution (LRS). The washed cells were then diluted in LRS to a concentration of 9 × 10^7^ CFU/1.8 mL for the rabbit study. The number of bacteria in the inoculum was confirmed by serial dilution on 5% sheep blood agar plates. MEDI3902 was expressed and purified as described previously (7).

### Animal investigation protocol.

The rabbit model of ventilator-associated pneumonia was reviewed and approved by the University of California, San Francisco, Institutional Animal Care and Use Committee and was conducted in a facility accredited by the Association for Assessment and Accreditation of Laboratory Animal Care International. Pathogen-free male New Zealand White outbred rabbits (16 to 20 weeks of age, 3.6 to 4.0 kg; Western Oregon Rabbit Company) were used in all animal studies. Rabbits were housed in single stainless-steel cages in a climate-controlled housing room with a daily 12-h light/12-h dark cycle. They were provided rabbit food pellets and water *ad libitum*, which were supplemented twice daily with hay, bananas, apples, lettuce, celery, and carrots.

### Experimental groups.

We used 8 rabbits for study 1, in which the animals were monitored for 36 hpi, and 8 rabbits for study 2, in which the animals were monitored for 60 hpi. For each of the two studies, 4 of the rabbits were randomized for prophylactic intravenous administration of 15 mg/kg MEDI3902 at 24 h before infection, whereas the other 4 rabbits were treated with 15 mg/kg R347, an irrelevant isotype c-IgG.

### Mechanical ventilation and aseptic surgery.

Rabbits were anesthetized by intramuscular administration of a freshly prepared mixture of 35 mg/kg ketamine and 5 mg/kg xylazine and then were intubated with a 3.0-mm pediatric endotracheal tube with the cuff inflated to 18 to 20 mm Hg. The endotracheal tube was connected to a veterinary anesthesia delivery ventilator (ADS2000; Engler). The pressure-controlled ventilator was set to deliver a peak inspiratory pressure (PIP) of 15 cm H_2_O, PEEP of 6 cm H_2_O, flow rate of 4 L/min, respiratory rate of 30 to 35 breaths/min, and FiO_2_ of 0.35, with 2.00% isoflurane to maintain general anesthesia (Fig. 1). A neonatal flow sensor with a dead space volume of <1 mL was connected to the endotracheal tube to measure baseline airway flow and pressure of the ventilated rabbits (IntelliVue patient monitor; Philips). The flow sensor was removed from the breathing circuit immediately after baseline measurement because it increased dead space volume, showing that the mechanical ventilation parameters resulted in a low tidal volume of 6 to 7 mL/kg and an inspiratory/expiratory ratio of 1:2.5.

Aseptic surgical techniques were used to cannulate the right carotid artery with an 18-gauge arterial line catheter, which was connected to a pressure monitoring kit with the Safedraw blood sampling system (Argon) for continuous invasive blood pressure monitoring and arterial blood sampling. The patency of these lines was maintained by continuous flushing with 0.9% saline containing 1 U/mL heparin in a 1,000-mL bag pressurized to 300 mm Hg.

For fluid replacement, the marginal ear vein was cannulated with a 22-gauge intravenous catheter for continuous infusion of Normosol-R with 5% dextrose at 2 mL/kg/h using an intravenous infusion pump (Hospira).

### P. aeruginosa infection.

After approximately 2 to 3 h of mechanical ventilation, the rabbit was injected with a mixture of 12 mg/kg ketamine and 1.7 mg/mL xylazine, and then a 1.8-mL bacterial inoculum was instilled directly into the lungs through the endotracheal tube. The rabbit was then placed in the right lateral decubitus position for the duration of the study. Rabbits were euthanized with an overdose of sodium pentobarbital while still under general anesthesia after the predetermined monitoring period of either 36 hpi (study 1) or 60 hpi (study 2) or after attainment of humane endpoints, which were defined as arterial oxygen saturation of <60% and arterial blood gas analysis showing lactate levels of >15.0 mmol/L and base excess less than −10.0 mmol/L.

### Longitudinal blood biomarker analyses.

Arterial blood samples were collected from the carotid artery at baseline (30 to 60 min before infection), every 2 h for the first 24 hpi, and then every 4 h for 28 to 60 hpi. Each blood sample was characterized for (i) 5-part WBC differential and red blood cell and platelet parameters using the Element HT5 veterinary hematology analyzer (Heska); (ii) partial pressure of CO_2_, partial pressure of O_2_, Na^+^, K^+^, Cl^−^, ionized Ca^2+^, creatinine, glucose, lactate, pH, and base excess using the Element POC rapid blood analyzer (Heska); and (iii) alanine aminotransferase, amylase, and creatinine levels using the Comprehensive Diagnostic Profile rotor with the VetScan VS2 system (Abaxis).

### LW/BW ratio and bacterial counts in organs and blood.

Lungs, spleen, and kidneys were removed aseptically from euthanized animals, weighed, and immediately processed. The right lung, spleen, and kidneys were cut into <0.5-cm pieces, and 0.2- to 0.3-g samples were homogenized in 0.9% saline, followed by quantification of CFU by serial dilutions on 5% sheep blood agar (Remel). Arterial blood collected every 2 to 4 h for biomarker analysis (see above) was diluted (100 μl of blood in 900 μl of sterile water) and plated for quantitative culture on 5% sheep blood agar (Remel).

### Lung inflammatory cytokine expression analysis.

Right lung pieces were preserved in RNAlater (Thermo Fisher Scientific) within 5 to 7 min after euthanasia. RNA was extracted, and differential expression of 84 genes encoding cytokines and receptors was then evaluated using the rabbit-specific inflammatory cytokines and receptors real-time reverse transcriptase (RT^2^) Profiler kit (PANZ-011ZA; Qiagen) as described previously (16). In brief, RNA was extracted from two RNAlater-preserved samples from each lung. The RNA integrity number (RIN) evaluation (RIN of >5), PCR array reproducibility, RT efficiency, and genomic DNA contamination were evaluated for each RNA extraction for quality control purposes. Average cycle threshold (*C_T_*) values were used for each tissue, using a threshold of 0.051. The mean *C_T_* values for four rabbit housekeeping genes, *ACTB*, *GAPDH*, *LDHA*, and *loc100346936*, were used for normalization. For each gene, the fold regulation was computed as the fold change in the lungs of rabbits pretreated with MEDI3902, relative to those pretreated with c-IgG, using normalized *C_T_* values.

### Histology.

The left lungs were inflated by gravity with 10% neutral buffered formalin, fixed for 72 h at 8°C, and then transferred to 70% ethanol. Fixed tissues were processed according to standard methods, as described previously (40), and stained with Gill’s hematoxylin (Mercedes Medical, Sarasota, FL) and eosin (Surgipath, Richmond, IL) for histological evaluation by a pathologist (L. I. Cheng) who was blinded to the experimental conditions.

### Statistical analysis.

The prespecified hypothesis was that MEDI3902 prophylaxis would be superior to c-IgG prophylaxis with respect to survival after infection in the rabbit model of ventilator-associated pneumonia. Based on the previously reported prophylactic efficacy of MEDI3902, compared to c-IgG, in a rabbit acute pneumonia model (16), we calculated that a sample size of four animals per experimental group in a rabbit ventilator-associated pneumonia model would provide a power of 90% to detect a hazard ratio of 0.10 favoring MEDI3902 over c-IgG, with a one-sided type I error of 0.05 by means of a log rank test (Schoenfeld method) using STATA software, version 10.0 (StataCorp). Survival curves were generated using the Kaplan-Meier method, and significance was assessed by means of the log rank (Mantel-Cox) test. Two-sided Fisher’s exact test was used to evaluate associations between bacteremia and prophylaxis with MEDI3902 or c-IgG. Normal distribution was not assumed; therefore, variables were compared using a nonparametric two-sided Mann-Whitney *U* test.

For analysis of gene expression data, a one-way analysis of variance (ANOVA) model with heterogeneous within-group variance showed no difference between the c-IgG groups used in the two rabbit studies (*P *> 0.05), thus allowing evaluation of fold gene regulation of MEDI3902-treated animals relative to the pooled c-IgG animals. The 95% confidence interval (CI) for fold regulation and the false-discovery rate (FDR)-adjusted *P* values are reported (16, 41).
