# Supplementary material for: Antivirulence Bispecific Monoclonal Antibody-Mediated Protection against Pseudomonas aeruginosa Ventilator-Associated Pneumonia in a Rabbit Model
Source: Antimicrob Agents Chemother. 2022 Feb 15;66(2):e02022-21. doi: 10.1128/aac.02022-21 (PMC8846318; doi:10.1128/aac.02022-21)
Supplement: Supplemental file 1 — Supplemental material. Download aac.02022-21-s0001.pdf, PDF file, 0.2 MB [file aac.02022-21-s0001.pdf]

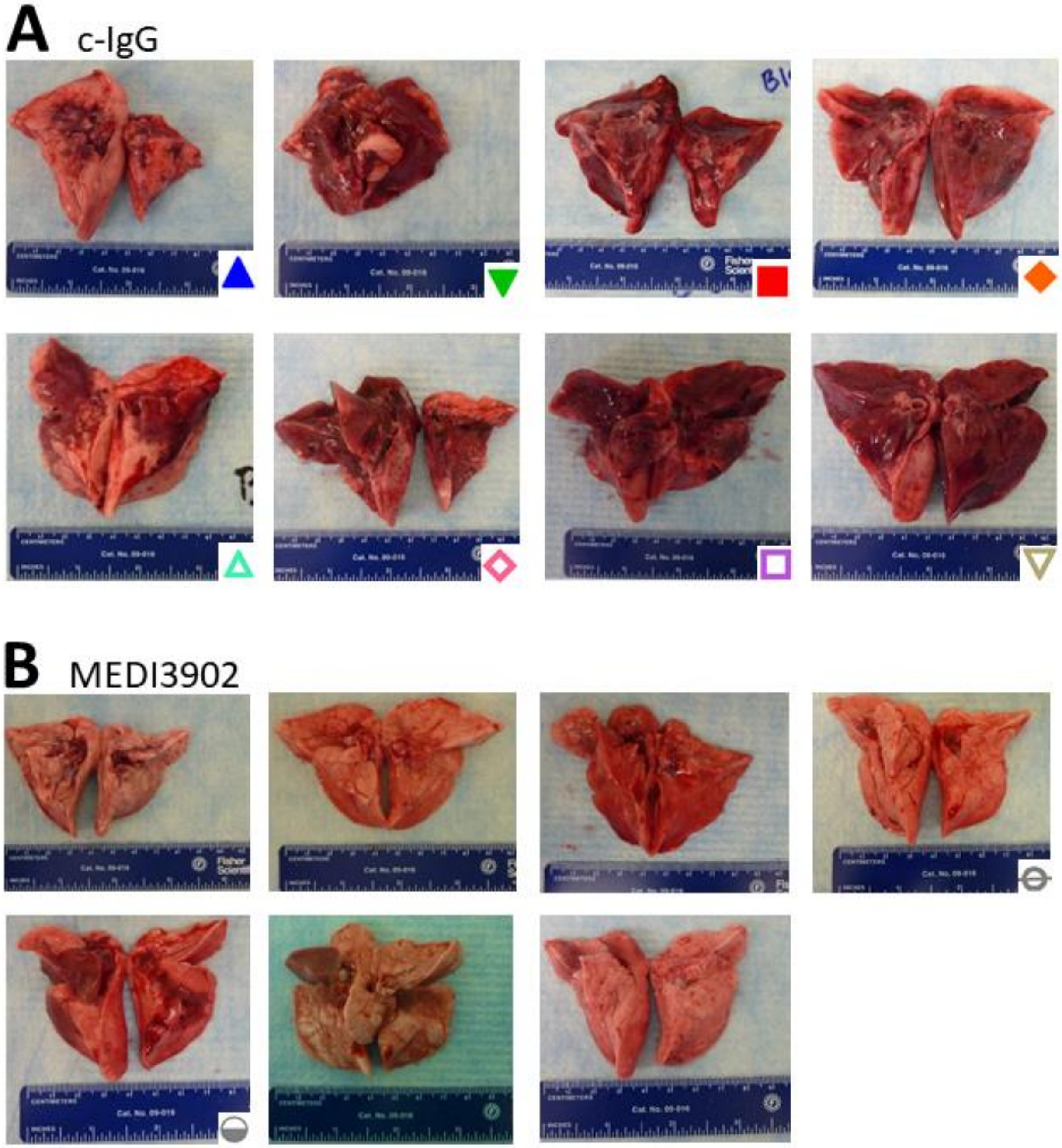

Supplemental Figure S1. Lungs from rabbits pretreated with (**A**) c-IgG at the time when they succumbed to infection, or (**B**) MEDI3902 at 36 h post infection or 60 h post infection. One lung image from a MEDI3902-pretreated rabbit is not available due to a camera malfunction. Symbols displayed in the lower right corner for selected lungs correspond to those used in **Figures 1,4,5**.

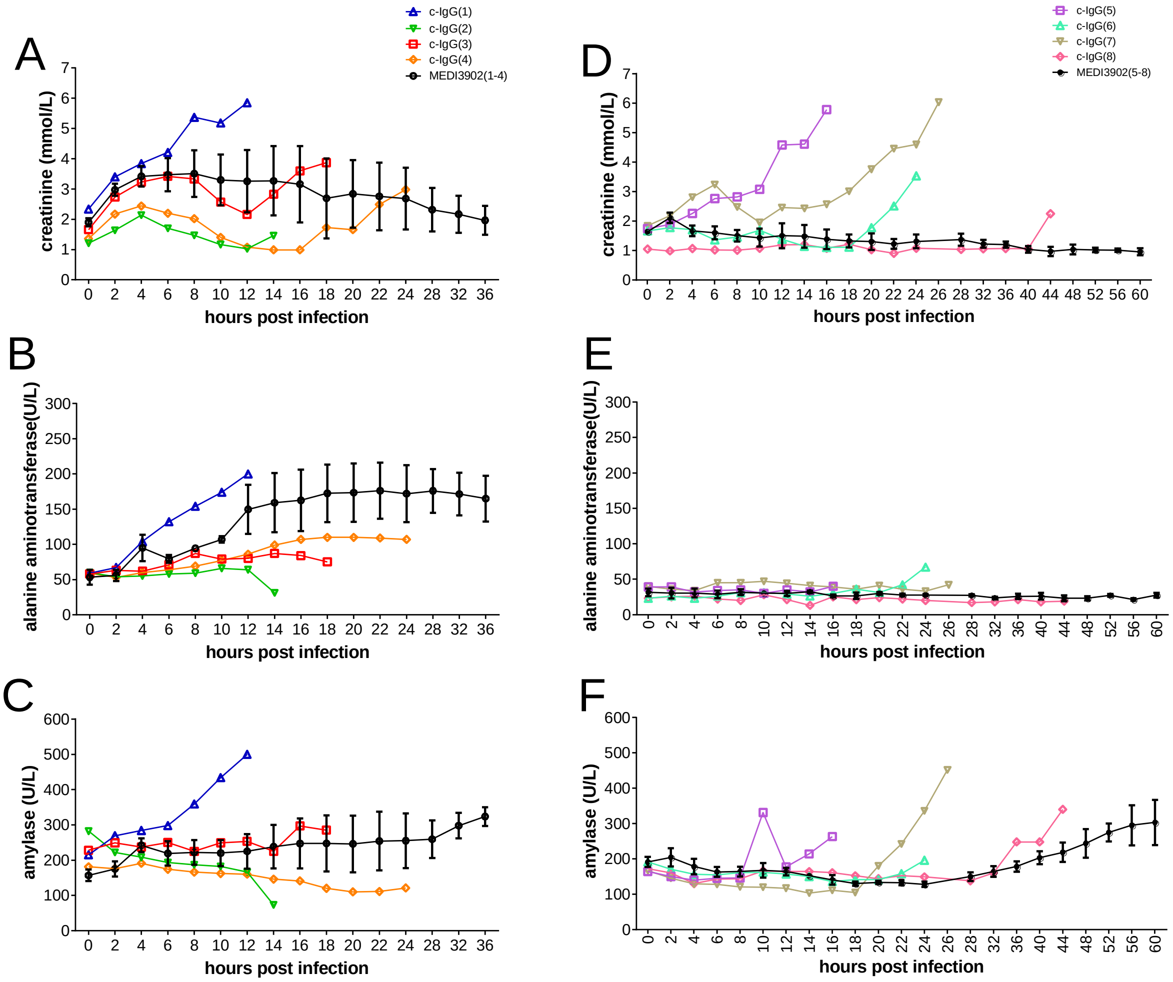

Supplemental Figure S2. Longitudinal changes in organ injury biomarkers for rabbits pretreated with MEDI3902 versus c-IgG. (**A**,**D**) creatinine, (**B**,**E**) alanine aminotransferase, and (**C**,**F**) amylase determined using arterial blood samples taken every 2 hours for the first 24 h and then every 4 h thereafter until survivors were euthanized at 36 hpi (**A-C**, Study 1) or 60 h (**D-F**, Study 2).

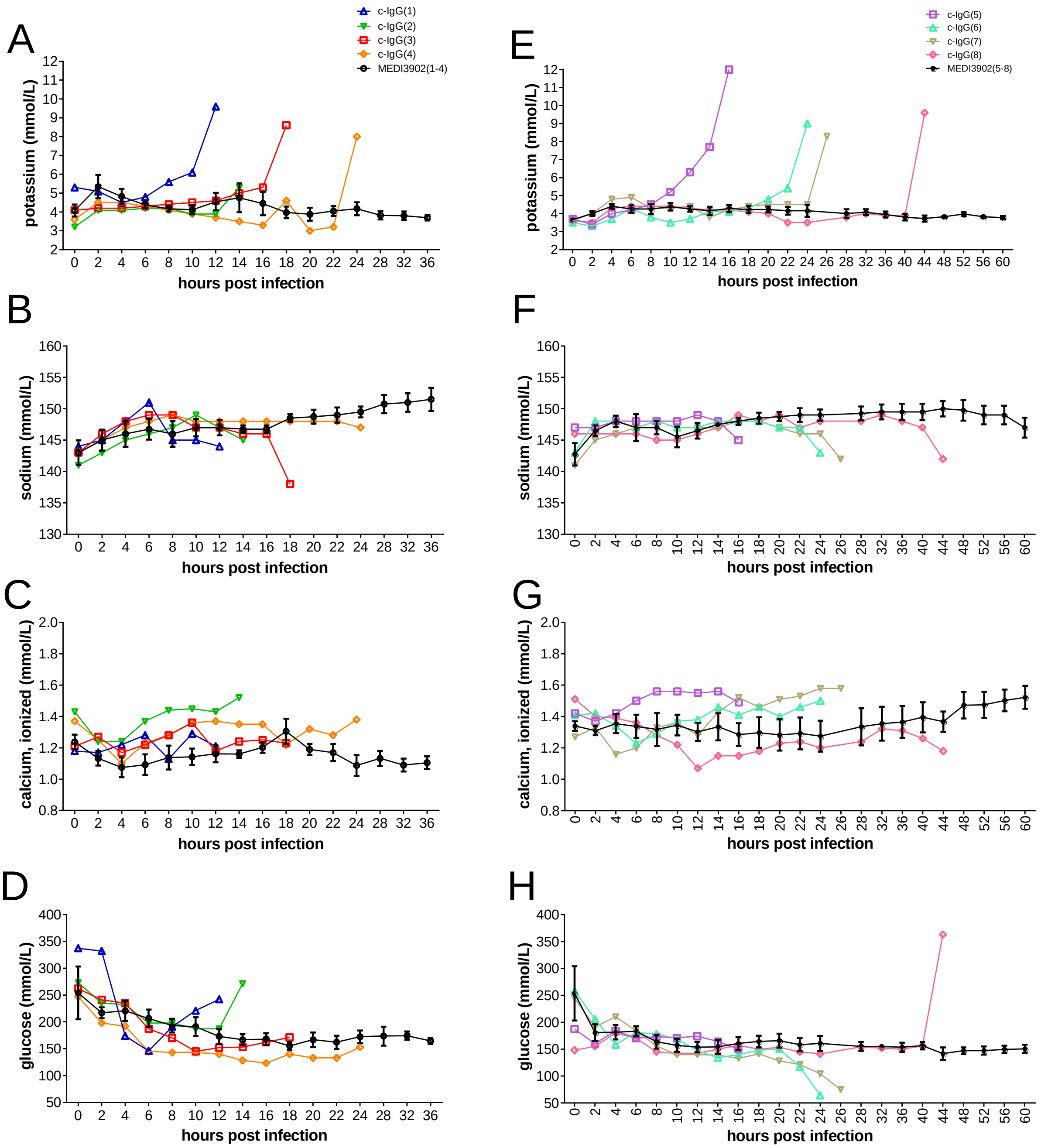

Supplemental Figure S3. Longitudinal changes in electrolytes and glucose for rabbits pretreated with MEDI3902 versus c-IgG. (**A**,**E**) potassium, (**B**,**F**) sodium, (**C**,**G**) calcium, and (**D**,**H**) glucose determined using arterial blood samples taken every 2 hours for the first 24 h and then every 4 h thereafter until survivors were euthanized at 36 hpi (**A-D**, Study 1) or 60 h (**E-H**, Study 2).

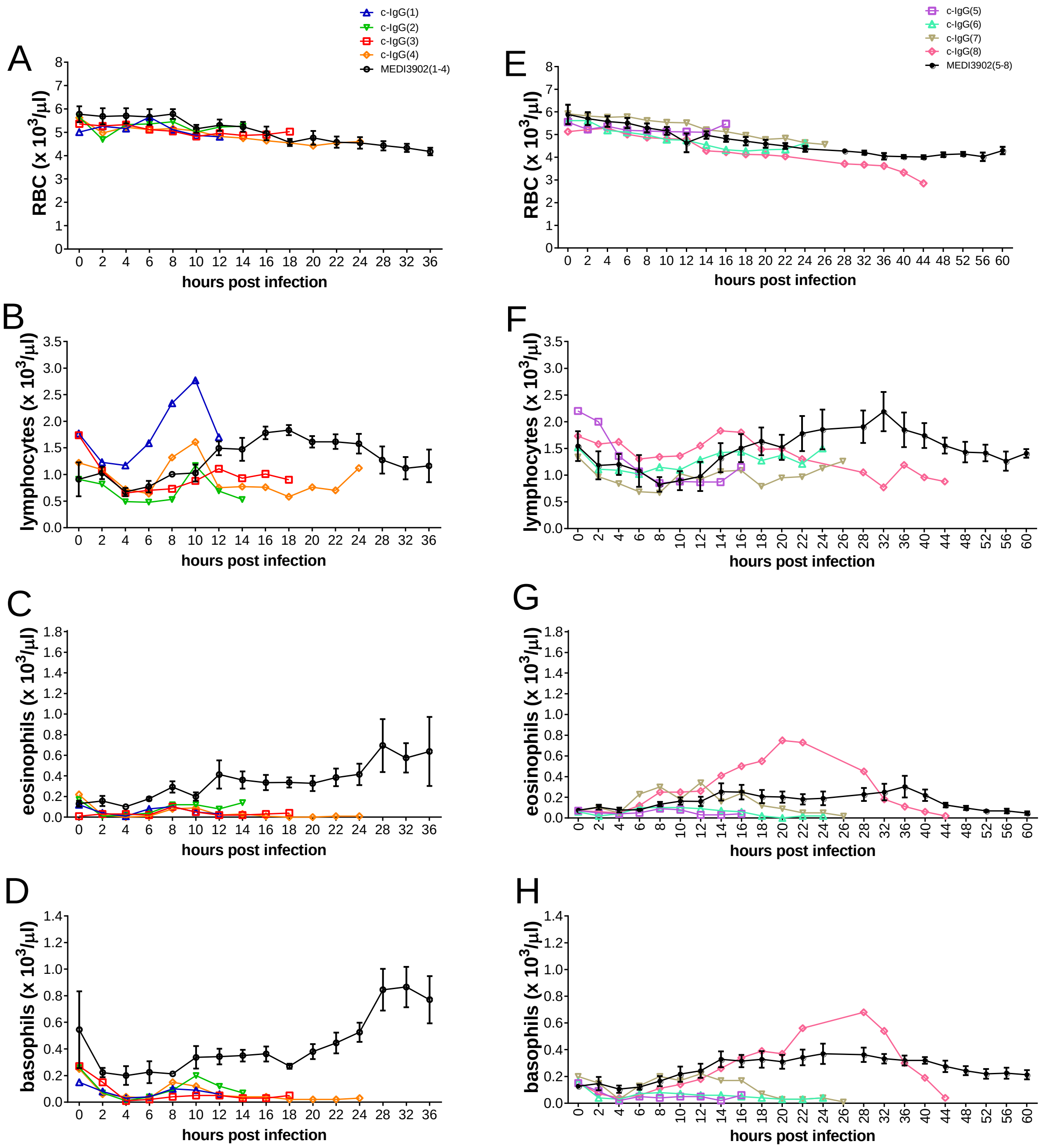

Supplemental Figure S4. Longitudinal changes in hematological parameters for rabbits pretreated with MEDI3902 versus c-IgG. (**A**,**E**) Red blood cells, RBC, (**B**,**F**) lymphocytes, (**C**,**G**) eosinophils, and (**D**,**H**) basophils determined using arterial blood samples taken every 2 hours for the first 24 h and then every 4 h thereafter until survivors were euthanized at 36 hpi (**A-D**, Study 1) or 60 h (**E-H**, Study 2).
